# An Algorithm for Strategic Continuation or Restriction of Asthma Medication Prior to Exercise Challenge Testing in Childhood Exercise Induced Bronchoconstriction

**DOI:** 10.3389/fped.2022.800193

**Published:** 2022-02-22

**Authors:** Vera. S. Hengeveld, Pascal B. Keijzer, Zuzana Diamant, Boony J. Thio

**Affiliations:** ^1^Department of Paediatrics, Medisch Spectrum Twente, Enschede, Netherlands; ^2^Department of Microbiology, Immunology and Transplantation, KU Leuven, Catholic University of Leuven, Leuven, Belgium; ^3^Department of Respiratory Medicine and Allergology, Institute for Clinical Science, Skane University Hospital, Lund University, Lund, Sweden; ^4^Department of Respiratory Medicine, First Faculty of Medicine, Charles University and Thomayer Hospital, Prague, Czechia; ^5^Department of Clinical Pharmacy and Pharmacology, University of Groningen, University Medical Center Groningen, Groningen, Netherlands

**Keywords:** asthma, ECT, ICS, medication, algorithm, EIB, precision medicine, pediatrics

## Abstract

Exercise induced bronchial (EIB) constriction is a common and highly specific feature of pediatric asthma and should be diagnosed with an exercise challenge test (ECT). The impact of EIB in asthmatic children's daily lives is immense, considering the effects on both physical and psychosocial development. Monitoring childhood asthma by ECT's can provide insight into daily life disease burden and the control of asthma. Current guidelines for bronchoprovocation tests restrict both the use of reliever and maintenance asthma medication before an exercise challenge to prevent false-negative testing, as both have significant acute bronchoprotective properties. However, restricting maintenance medication before an ECT may be less appropiate to evaluate EIB symptoms in daily life when a diagnosis of asthma is well established. Rigorous of maintenance medication before an ECT according to guidelines may lead to overestimation of the real, daily life asthma burden and lead to an inappropiate step-up in therapy. The protection against EIB offered by the combined acute and chronic bronchoprotective effects of maintenance medication can be properly assessed whilst maintaining them. This may aid in achieving the goal of unrestricted participation of children in daily play and sports activities with their peers without escalation of therapy. When considering a step down in medication, a strategic wash-out of maintenance medication before an ECT aids in providing objective support of potential discontinuation of maintenance medication.

## Key Messages

1. In addition to chronic effects, maintenance asthma medications also have acute bronchoprotective effects against exercise induced bronchoconstriction (EIB).
2. An Exercise Challenge Test (ECT) with continuation of daily maintenance medications represents real-life disease burden and protection against EIB and thus can support personalized decision making.
3. A strategic wash-out of maintenance medication before an ECT can support a safe step-down in maintenance medication in children with exercise-induced bronchoconstriction.

## Introduction

Asthma is the most common chronic inflammatory disease in childhood, affecting up to 10% of all children and impairing quality of life (1, 2). Exercise-induced bronchoconstriction (EIB) is a common and a highly specific symptom of childhood asthma which frequently persists despite maintenance treatment with maintenance medications. It is a sign of bronchial hyperresponsiveness (BHR) due to airway inflammation, indicative of poor asthma control (3–6). In children with EIB, exercise-induced hyperpnea triggers the release of mediators from inflammatory cells residing in the airway lining. This results in airway smooth muscle contraction, congestion of the airway lining and increased mucus production, all contributing to narrowing of the airway (7–10).

Although EIB commonly presents with the classic symptoms of childhood asthma, i.e. coughing, wheezing and dyspnea during or after exercise, symptoms can be subtle or less specific and can overlap with other common causes of exertional dyspnea. EIB restricts activity while playing and during sports due to exercise limitation (11, 12). Considering the impact of activity on both physical and psychological development of children, early identification and treatment of childhood EIB is essential (13–16).

Prior studies have shown that questionnaires cannot accurately predict the presence and severity of EIB in children (17–22). The diagnosis of EIB should thus always be accompanied by documentation of changes in lung function in response to an exercise (or a surrogate) challenge test (23–25). In this context, we advocate the use of an exercise challenge test (ECT), as it simulates the real-life situation in which relevant symptoms usually occur as well as the fact that other challenge tests may be negative even in the presence of EIB (26–28). An ECT also allows diagnosing other disorders that can mimic EIB, e.g., dysfunctional breathing or exercise induced laryngeal obstruction (EILO) (29, 30). A fall in forced expiratory volume in one second (FEV_1_) of more than 13% after exercise provocation is generally considered diagnostic for EIB (5, 23, 24, 31).

Here we discuss implications of (dis)continuation of EIB therapy before an ECT in patients based on the mechanism of action of the medications and real-life experience. Additionally, we propose an algorithm to (dis)continuation of anti-EIB medications to enable correct and practical interpretation of ECT outcomes in the individual patient.

## Asthma Medication and EIB

Pharmacological treatment of significant childhood EIB consists of reliever [e.g., short-acting β2-adrenoreceptor agonists (SABA)] and maintenance medication [e.g., inhaled corticosteroids (ICS) or leukotriene receptor antagonists (LTRA)] (32). Sometimes add-on maintenance therapies such as long-acting β2-adrenoreceptor agonists (LABA), LTRA, long-acting muscarinic antagonists (LAMA) and/or antihistamines are necessary to achieve the goals of EIB management, i.e., symptom control, exercise tolerance and participation in play and sports (23, 32).

The occurrence and severity of EIB as reported by patients or measured during an ECT is strongly influenced by acute and chronic effects of prescribed reliever and maintenance medications, respectively. Table 1 shows medication withholding times before an ECT as advised in the current ERS technical standard on bronchial challenge testing (25). For correct interpretation of ECT results, it is important to be aware of the magnitude and duration of protection against EIB of the different types of asthma medications. We will shortly summarize the anticipated effects of commonly prescribed asthma medications on EIB, before elaborating on strategic continuation or restriction of specific medication before an ECT to aid clinical decision making in individual patients.

**Table 1:** Withholding Times Prior to Indirect Challenge Testing According to ERS Technical Standard on Bronchial Challenge Testing in Children.

**Medication**	Recommended withholding time before challenge test *
SABA (salbutamol)	8 h
LABA (salmeterol, formoterol)	36 h
LABA + ICS (salmeterol/fluticasone propionate, formoterol/budesonide)	36 h
Ultra-LABA + ICS (vilanterol/fluticasonfuroate)	48
ICS (budesonide, fluticasone propionate, beclomethasone)	6 h
Long-acting ICS (fluticasone furoate, ciclesonide)	24
LTRAs (montelukast)	4 d
Antihistamines (loratadine, cetirizine)	72 h
LAMA (tiotropium bromide)	72 h

*Examples of specific medications within the class are provided in parenthesis. SABA, short-acting β2-adrenoreceptor agonists; LABA, long-acting β2-adrenoreceptor agonists; ICS, inhaled corticosteroids; LTRA, leukotriene receptor antagonists; LAMA, long-acting muscarinic antagonists*.

* *As advised by the ERS technical standard on bronchial challenge testing (23, 26)*.

### Reliever Medication

SABA's exert their bronchodilator effects via β2-adrenoreceptors located on airway smooth muscle cells. Activation of these receptors results in smooth muscle cell relaxation resulting in airway dilation. SABA's can be taken to relieve EIB once it has occurred, but are preferably administered pre-exercise to prevent EIB. Additionally, β2-adrenoreceptors are also expressed on inflammatory cells including mast cells, macrophages and eosinophils as well as on structural cells: i.e., submucosal glands, vascular endothelium and vascular smooth muscle cells. These locations further facilitate the bronchoprotective effects of SABA's (33).

SABA's have a rapid onset of action with a duration lasting for 4 to 6 h (34). SABA's nearly blunt EIB when administered pre-exercise, reducing the fall in FEV1 by 70–80% in most patients (35, 36). Guidelines advise a withholding time of 8 h prior to an ECT (Table 1). In contrast to their acute bronchodilating and bronchoprotective effects, prolonged and/or frequent use of SABA's induces tolerance due to receptor desensitization (33). This tolerance manifests as a reduced duration of protection (2 h), a more rapid onset of EIB and a slow and incomplete response to rescue treatment with SABA's (36, 37).

### Maintenance Medication

ICS are the cornerstone of maintenance treatment for asthma and EIB in children (aged 6 to 11), although the updated GINA document recommends combination therapy (ICS + LABA) as step 1 maintenance treatment in adolescents (aged 12 and above) (32). Corticosteroids possess anti-inflammatory properties which account for their effectiveness in suppressing the underlying airway inflammatory process and controlling EIB symptoms. ICS provide 50–60% protection against EIB (38–41) and reach their maximal effect within 3 weeks of maintenance treatment, although efficacy may vary across brands and among patients (42).

Besides these chronic effects, previous studies have also shown acute protection against EIB after administration of only a single dose of ICS (43–46). Visser et al. demonstrated a protection ≥50% against EIB in the majority of children (aged 5–16) 4 h after a single low dose of ICS (44). In adult asthmatics, acute positive effects on lung function of a single high dose ICS have been shown to last for at least 8 to 9 h (47). Current guidelines however advise to withhold ICSs for only 6 h prior to an ECT (Table 1) which may incompletely nullify acute effects of ICS (25).

LABA's act on β2-adrenoreceptors similar to SABA's, but have a prolonged duration of action compared to SABA's. Similar to SABA's, LABA's induce tolerance and are therefore recommended not to be used as monotherapy but in combination with ICS that mitigate potentially adverse inflammatory consequences of chronic β2-agonist therapy (48). Inhalation of a single dose of salmeterol produced both a statistically significant and a clinically important bronchodilator and bronchoprotective effect for at least 12 h in children aged 6 to 12 (49). Similar effects have been found in adults using formoterol (50). The guidelines advise to withhold LABA's for 36 h prior to an ECT (Table 1) (25).

LTRA's are commonly used in childhood asthma as (add-on) maintenance therapy. LTRA's block the cysteinyl leukotriene receptor, inducing anti-inflammatory effects. Cysteinyl leukotrienes are pro-inflammatory mediators released from activated mast cells and eosinophils that cause potent and long-lasting airway narrowing (51, 52). Apart from modest increases in baseline FEV1, LTRA's inhibit the maximal bronchoconstrictor response after exercise and shorten the time to recovery to pre-challenge lung function (53, 54). In their cross-over study, Kim et al. even showed persistent significant improvement of asthma symptom score, maximum post-exercise fall in FEV1 and time to recovery as long as 8 weeks after stopping montelukast (54). Besides chronic effects against EIB, placebo-controlled studies in children (aged 4–14 years) also showed an acute bronchoprotective effect 2 h after a single oral dose of montelukast that persisted up to 24 h (55). Current guidelines advise to withhold LTRA's 4 days prior to an ECT (Table 1) (25).

LAMA's are included in the GINA 2021 guidelines as optional add-on maintenance therapy in both children and adolescents whose asthma remains uncontrolled despite treatment with ICS-LABA (32). LAMA's act by blocking muscarinic acetylcholine receptors on the airway smooth muscle cells, causing airway relaxation (56, 57). Inhibition of these muscarinic receptors may also play a role in reducing mucus secretion, inflammation and airway remodeling, thereby leading to reduced airway hyperresponsiveness (56, 58). Tiotropium has shown to improve lung function as add-on therapy for both children (aged 6–11 years) and adolescents with moderate-to-severe symptomatic asthma despite ICS (and 1 or more controller medications) (59–62). Blais et al. furthermore demonstrated a significant bronchoprotective effect of inhalation of a single dose tiotropium or glycopyrronium in adults (measured by metacholine provocation), that for tiotropium lasted up until 7 days after administration of the single dose. There is no literature on the effect of LAMA's on bronchoprovocation by indirect challenge tests such as an ECT (63). Guidelines currently advise to withhold LAMA's for at least 72 h prior to an ECT (Table 1) (25).

## Continuation or Restriction of Medication Before an ECT – *That's the Question*

**Figure 1:**
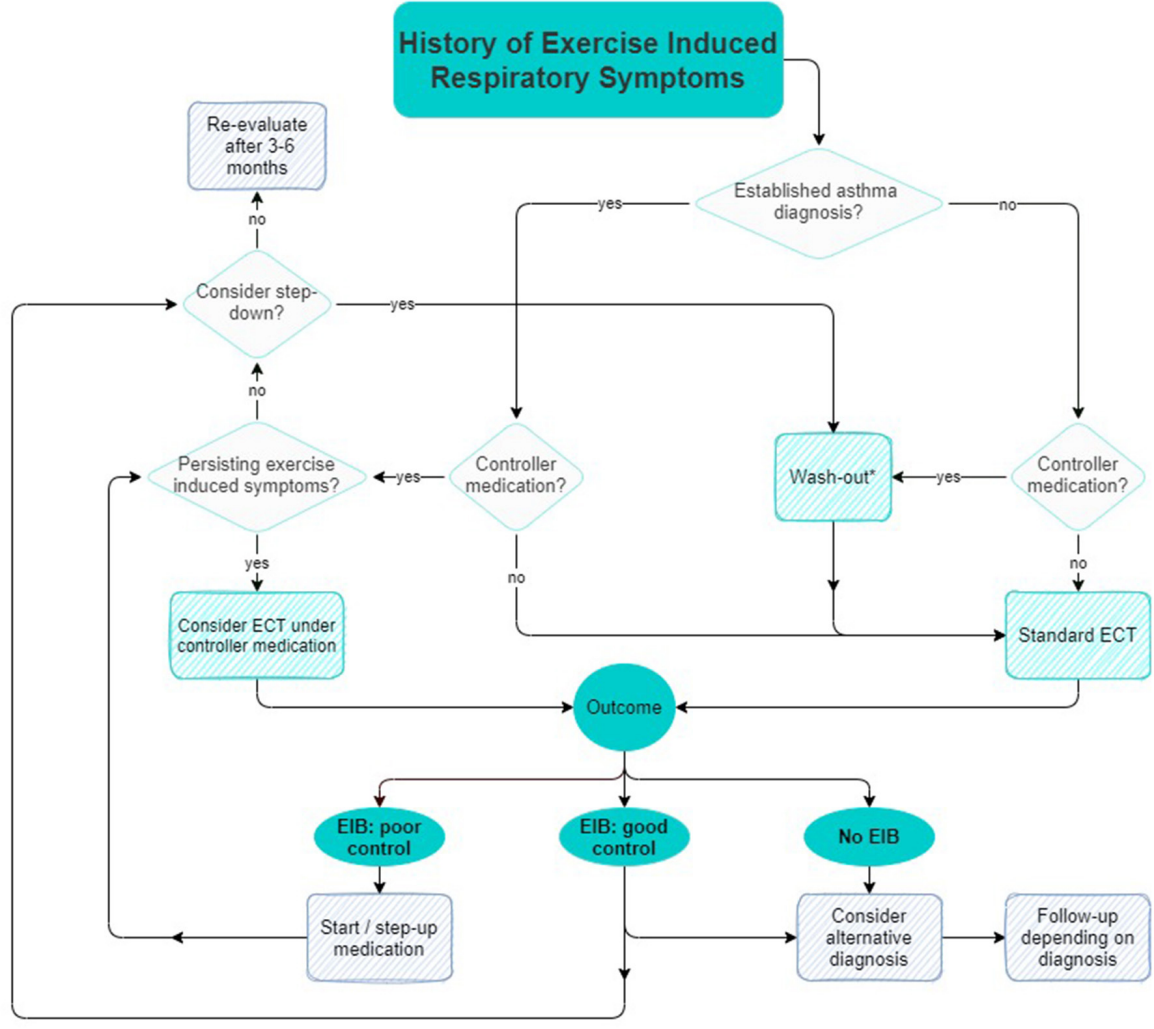
Suggested algorithm for strategic continuation or restriction of asthma medication prior to an ECT.

The technical standard on indirect provocation tests states that all medication should be withheld to prevent false-negative tests and ensure proper diagnosis (23, 25). However, advice regarding withholding times of medication may become impractical, as medication regimes often contain multiple types of anti-EIB medication with different withholding times as listed in Table 1. This easily leads to incorrect restriction of medication by patients in real-life settings. Since asthma medication has both acute and chronic bronchoprotective- and/or bronchodilator effects, continuation or restriction of medication significantly affects EIB occurrence and severity as assessed by an ECT. Figure 1 shows a suggested algorithm regarding strategic continuation or restriction of asthma medication before an ECT. The goal of this algorithm is to support personalized medicine by preventing under- or overtreatment of EIB in individual patients. Future studies could validate the efficacy of this algorithm in preventing inappropriate escalation of therapy and managing controlled step-down in medication.

### Establishing Asthma Diagnosis

When a diagnosis of asthma is uncertain while maintenance medication has been started, a ‘blank' diagnostic test after a full wash-out of maintenance medication can be useful. Previous studies suggest that a wash-out of at least 2 weeks is appropriate to diminish chronic bronchoprotective effects of maintenance medications (64). A negative ECT after a full wash-out of maintenance medication indicates absence of EIB. In case medication is only restricted for a shorter period of time according to the guidelines, a negative EIB test could be compatible with either a good EIB control, as well as absence of EIB, as the example in Box 1 describes. Establishing a proper diagnosis before treatment prevents overtreatment and allows clinicians to objectively follow-up on therapy effects.

Box 1 ***Case 1* “*Is this EIB?*”**

*A high-performing 17 years old female soccer player was referred to our pediatric clinic with a history of exercise-induced symptoms and wheezing for which she received ICS for 2 years, as prescribed by her general practitioner. No other asthma symptoms or signs were apparent. She was referred for an ECT because of persisting exercise-induced symptoms. An ECT was performed after a 2 weeks wash-out of her ICS. The ECT showed no EIB but was suggestive of a mild exercise-induced laryngeal obstruction (EILO), for which she was referred to a speech therapist*.

### Persisting Exercise-Induced Respiratory Symptoms

An objective follow-up of EIB control is especially useful as it is not uncommon for (a step-up of) maintenance medication to reduce but not completely suppress EIB. An ECT has a high degree of within subject repeatability and can therefore be used to evaluate asthma therapy effectiveness (21, 22). In case of persistent exercise-induced respiratory symptoms despite adequate(ly taken) maintenance therapy, there are two diagnostic possibilities. Firstly, symptoms could be due to inadequate asthma control under current medication, usually requiring a step-up in medication. Secondly, the persisting symptoms could be of a non-asthmatic origin. Other disorders such as EILO, dysfunctional breathing or a poor cardiopulmonary condition often coexist with EIB and can mimic EIB symptoms (30, 65, 66). When persisting respiratory symptoms are not caused by EIB, a step-up in medication would be both ineffective and detrimental as this would delay appropriate therapy, as described by the example in Box 2.

An ECT under continuation of daily life medication regime can be helpful to evaluate causes of daily life symptoms, disease burden and bronchoprotection, supporting personalized decision making in individual patients.

### Considering Step-Down

When symptoms of EIB have been controlled for 3 months and lung function improvement has reached a plateau, a step-down in medication can be considered. GINA advises to consider any step-down in asthma medication as a therapeutic trial, where response should be evaluated in terms of both symptom control and exacerbation frequency (32). An ECT two weeks after a step-down facilitates a full wash-out of acute and chronic effects of maintenance medication and can be used to objectively evaluate symptom control of EIB and to support a controlled step-down (64).

Box 2 ***Case 2* “*Evaluating necessity of step-up*”**

*A 9-year old-boy was under control in our pediatric clinic because of asthma and allergic rhinitis. He used a high dose ICS* + *LABA and an LTRA in order to control his asthma. Despite this therapy, he had several exacerbations in the past years, exercise intolerance and frequent SABA use. There was doubt about his therapy adherence/technique regarding his maintenance medication and/or if his current medication was insufficient to control his asthma. He was scheduled for an ECT and stopped his asthma medication according to guidelines, however pre-exercise spirometry showed an obstructive curve with an FEV1*<*70% of predicted and spirometer-induced bronchoconstriction requiring cancellation of the ECT. An ECT with continuation of ICS* + *LABA and LTRA was rescheduled to assess daily life symptoms and bronchoprotection under current medication. ICS* + *LABA were administered under supervision before his ECT, ensuring adherence and adequate technique. FEV1 was 92% of predicted, with a 7% fall in FEV1 post-ECT. Reported symptoms of dyspnea were provoked, which were due to dysfunctional breathing and a poor cardiopulmonary condition. His medication proved to provide adequate daily life bronchoprotection against EIB and was therefore continued, with a focus on education, therapy adherence and inhalation technique. He was also referred to a physiotherapist for breathing exercises*.

## Conclusion

EIB is a highly specific and persistent symptom of childhood asthma indicating poor disease control and negatively affecting patients' quality of life. The occurrence and severity of EIB as measured during an ECT is strongly influenced by both the acute and chronic bronchoprotective- and dilating effects of reliever and maintenance medication. Guidelines regarding restriction of asthma medications before an ECT are difficult to implement in real life and do not always support the clinician to adjust EIB treatment in individual patients. Here we suggest a practical algorithm for strategic continuation or restriction of maintenance medications before an ECT to support personalized decision making in individual patients.

## Data Availability Statement

The original contributions presented in the study are included in the article/supplementary material, further inquiries can be directed to the corresponding author.

## Author Contributions

PK and VH contributed equally to this work. All authors contributed to the article and approved the submitted version.

## Author Disclaimer

All views expressed within this manuscript are original and based upon findings of the authors. None of the views discussed represents standard practice at the above listed institutions.

## Conflict of Interest

In the past 3 years, ZD acted as Research Director at QPS-NL, an institution which received research support from several bio-pharmaceutical companies, esp within respiratory: HAL Allergy, Foresee Pharmaceuticals, Patara Pharma (now Respivant), Novartis. Furthermore, ZD received honoraria or speaker fees serving on advisory boards or as a consultant from: ALK, Antabio, AstraZeneca, Boehringer Ingelheim, GlaxoSmithKline, HAL Allergy, Merck Sharp &amp; Dohme, Sanofi-Genzyme-Regeneron, all outside the submitted work. The remaining authors declare that the research was conducted in the absence of any commercial or financial relationships that could be construed as a potential conflict of interest.

## Publisher's Note

All claims expressed in this article are solely those of the authors and do not necessarily represent those of their affiliated organizations, or those of the publisher, the editors and the reviewers. Any product that may be evaluated in this article, or claim that may be made by its manufacturer, is not guaranteed or endorsed by the publisher.
